# Impact of the Preparation Method on the Formulation Properties of Allantoin Hydrogels: Evaluation Using Semi-Solid Control Diagram (SSCD) Principles

**DOI:** 10.3390/gels10010058

**Published:** 2024-01-12

**Authors:** Robert-Alexandru Vlad, Teodora-Cătălina Dudici (Vlăgea), Muhammad Ali Syed, Paula Antonoaea, Emöke Margit Rédai, Nicoleta Todoran, Cornelia-Titiana Cotoi, Magdalena Bîrsan, Adriana Ciurba

**Affiliations:** 1 Pharmaceutical Technology and Cosmetology Department, Faculty of Pharmacy, George Emil Palade University of Medicine, Pharmacy, Science and Technology of Targu Mures, 38th Gheorghe Marinescu Street, 540142 Targu Mures, Romania; 2 Department of Pharmaceutical Sciences, Faculty of Chemistry and Life Sciences, Government College University Lahore, Lahore 54000, Pakistan; 3 Faculty of Pharmacy, Department of Drug Industry and Pharmaceutical Biotechnology, University of Medicine and Pharmacy “Grigore T. Popa”, 16 Universităţii Street, 700115 Iasi, Romania

**Keywords:** xanthan gum, allantoin hydrogel, rheology, semi-solid control diagram (SSCD), good quality index (GQI), stirring, parametric index (PI), parametric profile index (PPI)

## Abstract

Allantoin possesses numerous beneficial properties for the skin, like anti-irritant effects, wound healing, skin hydration, and epithelization. In this paper, we investigated a suitable preparation method for an allantoin hydrogel using the Semi-Solid Control Diagram (SSCD) method and characterized its rheological and consistency behavior. To accomplish this, xanthan gum (XG) was selected as a model gelling agent. Briefly, four hydrogels were prepared, two without allantoin (coded M01 and M02) and two with allantoin (M1 and M2). Similarly, the formulations were either prepared through magnetic stirring (M01 and M1) or homogenization in a mortar (M02 and M2). The prepared hydrogels were evaluated using the SSCD for specific parameters and indexes. The Good Quality Index (GQI) shows a higher value for the formulation, M1 = 6.27, compared to M2 = 5.45. This result is also underlined by the value of M01 = 6.45, which is higher than M02 = 6.38. Considering the consistency, the formulation M01 possessed the highest spreadability, followed by M02 and then the allantoin hydrogels M1 and M2. The rheological behavior had a thixotropic pseudoplastic flow for all the formulations. The use of SSCD pictographs outlined the rheological properties that need improvement, the method that is suitable to prepare the allantoin hydrogels, and the influence of the allantoin suspended in the XG hydrogel.

## 1. Introduction

Hydrogels represent a unique biomedical material that contains polymers capable of absorbing water, increasing their three-dimensional polymeric network [1]. Numerous active pharmaceutical ingredients (APIs) have been successfully incorporated into hydrogel matrices to improve skin permeation or to obtain the controlled release of the drug through the swollen hydrogels alongside a better biocompatibility of the dosage form [2,3,4,5,6,7,8].

**Figure 1:**
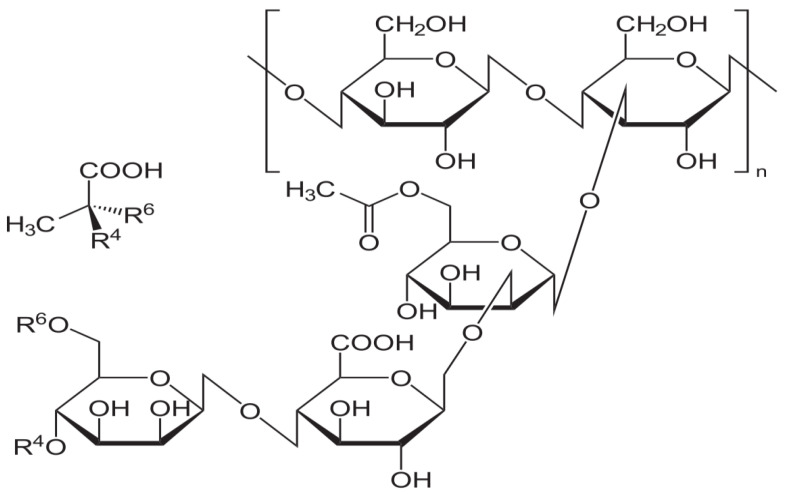
Xanthan gum’s chemical structure.

The hydrogel properties are chosen based on the selection of natural/synthetic gelling substances. Second to cellulose-based hydrogels, gums have also been proven successful in the fabrication of hydrogels for transdermal delivery [9]. Gums, belonging to the class of complex carbohydrates, have been incorporated as part of hydrogel components to deliver active substances as they possess controlled release characteristics, suitable rheological properties and flowability, easy extraction and processing for utilization, biocompatibility, and biodegradability [9,10]. Xanthan gum (XG), a short-branched non-exudate, obtained by fermenting sugar with *Xanthomonas* Sp., is a non-ionic, water-soluble, and non-toxic natural excipient (its structural formula is shown in Figure 1). It has applications in food, cosmetics, and pharmaceuticals as a thickening agent, stabilizer, gelling, and emulsifying agent [11,12]. When in contact with a hydrophilic environment, XG absorbs a large amount of water and forms interlinked polymeric chains [13]. XG has been effectively explored and uniquely designed to deliver active therapeutic substances as a transdermal membrane for diltiazem chloride [14], buccal mucoadhesive administration for periodontal therapy [15,16], stabilized gold nanoparticles [17], and controlled-release injectables [18]. A recent study reported the nano-capsule delivery of diphenyl diselenide consisting of a 3% (*w*/*v*) XG transdermal hydrogel used for the treatment of resistant melanoma and revealed a non-Newtonian flow of the gel at a pH value of 7. The nano-based hydrogel had a superior permeation through the dermis region [19]. Nevertheless, the adhesive properties of transdermal thermosensitive hydrogels containing two different types of Poloxamer (PF68 and F127) were enhanced by 1.24 and 1.64 times, respectively, when 0.2% (*w*/*w*) XG was added to the individual thermosensitive polymer hydrogel formulations as compared to formulations not containing XG [20]. These findings revealed that XG is a suitable component of hydrogels with desirable characteristics for transdermal delivery. The transdermal route is considered appropriate for site-specific and targeted drug delivery, especially when local action, for instance, healing, is desired. 

**Figure 2:**
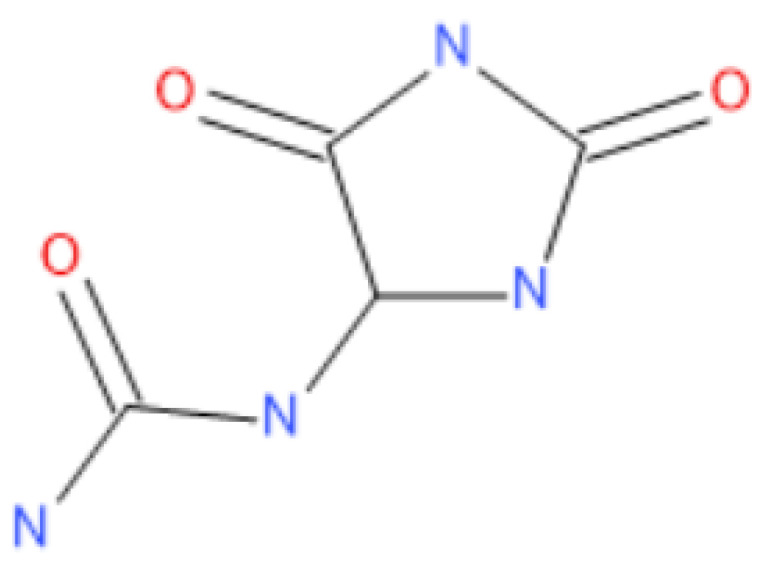
Allantoin’s structural formula.

Allantoin is a substance that can be used as adjuvant therapy for wound healing and for the treatment of dry scaly skin and hypertrophic scars. Moreover, a recent study on ethanol-induced gastric ulceration in rats demonstrated that allantoin-containing chitosan-based nanoparticles can also promote ulceration healing [21]. Chemically, it is a naturally occurring heterocyclic purine derivative and appears as imidazolidine dione in nature (5-ureidohydantoin) (Figure 2). It is an isotropic substance that shares some functional properties with hydantoin [22]. As a moisturizer, it is usually part of topical over-the-counter cosmeceutical products, such as sunscreen lotions, anti-acne products, deodorants, astringents, aftershave, and hand sanitizers. Likewise, it is frequently employed as an ingredient in buccal cavity products, like mouthwash, shampoos, and lipsticks [23,24]. Physically, it is a white crystalline powder, soluble in water at room temperature, with a solution pH in the range of 4.5–6 [24]. Its therapeutic effect has been proven in animals, where an allantoin-containing pectin hydrogel was applied topically on the skin of surgically incised wounds in Wistar rats. After the gel was applied for the healing process, the contraction in the wound region was observed along with a decreased healing time of almost 71%, suggesting that a pectin-based allantoin hydrogel is an effective way to treat skin wounds [25]. The dermal penetration and cell distribution of allantoin were increased when it was delivered into liposomal structures loaded with argan oil. The stability of the nano-based liposomal formulation revealed the stable diameter of the vesicles (less than 100 nm) [26]. The stability of the pharmaceutical semi-solid formulation is an important parameter that reflects the physical appearance of the formulation. Many topical formulations on the pharmaceutical/cosmetical market include 1% allantoin. This study aims to develop gel bases that are compatible with other products, such as enzymes, and that have very good stability or application qualities [21,22,23,24,25,26].

Allantoin is one of the only ingredients with excellent tolerance on the skin, having an anti-inflammatory action. It is fully compatible with cosmetic ingredients and with anionic, non-ionic, and cationic systems. Allantoin is suitable for any personal care application [23,24,25,26].

Xanthan gum is stable in applications with a wide range of pH values (2–12). It has a tolerance to enzymes, salt, and heat. The association between a mold-forming ingredient well tolerated by the skin and allantoin, a top ingredient in soothing and anti-acne or post-scarring products, is a benefit pursued through the development of a new gel base.

A mathematical tool known as the Semi-solid Control Diagram (SSCD) was developed as a measure for the quantitative scoring of the physical stability/pharmacotechnical parameters of semi-solid formulations. SSCD is a SeDeM-derived method used to optimize semi-solid formulations (gels, creams, and ointments) [27]. The SSCD evaluation is an effective strategy to estimate and compare the quality characteristics of the formulations developed. It is equally effective to compare and analyze the variations in inter-batch formulations prepared in the industry or the compounding lab at the hospital/pharmacy. This method has a unique propensity that can not only compare the physical evaluation of different ranges of semi-solid formulations, but from the pictorial presentation of the score of the parameters, we can also compare the related properties that may cause or risk instability.

**Figure 3:**
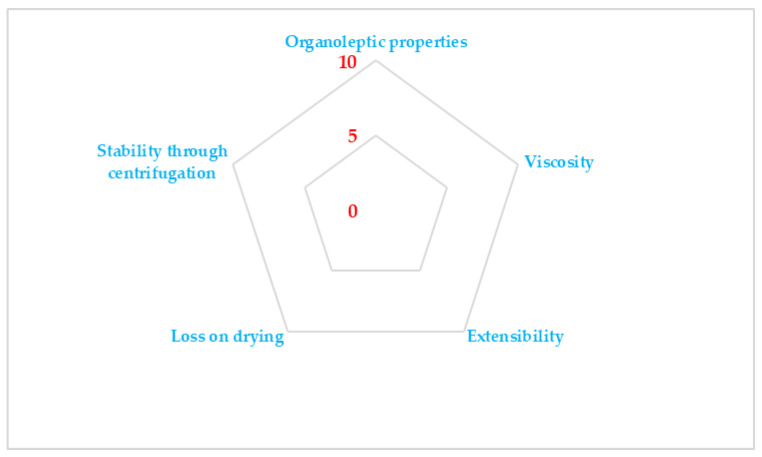
SSCD for semi-solid formulations.

SSCDs are traced using the Microsoft Office Excel tool. In this case, on each peak, five different properties are outlined, all of them important whilst developing semi-solid formulations (organoleptic properties, spreadability, viscosity, loss on drying, and stability through centrifugation) (an example of a SSCD is shown in Figure 3). Whilst for the last four, only one experiment is conducted, in the case of organoleptic properties, five different experiments are considered (homogeneity, color, flow through a cannula, air absence, and texture). Each of the five properties (organoleptic properties, spreadability, viscosity, loss on drying, and stability through centrifugation) receives a radius value in the range of 0–10 (0 is the lowest value and 10 is the maximum value). The targeted radius is at least five (values higher than five being recommended). For the experiments to assess the organoleptic properties’ parameters (homogeneity, color, flow through a cannula, air absence, and texture), values in the range of 0–2 are awarded and cumulated. If all the properties included in the organoleptic properties’ evaluation receive the maximum value, the radius for this parameter is 10. After the radius evaluation, three different indexes are calculated: Parameter Index (PI > 0.5), Parameter Profile Index (PPI > 0.5), and Good Quality Index (GQI > 5). Through these indexes, it can be established if the formulation corresponds to the pre-established quality requirements and it can be also noticed which parameter needs improvement. Also, the SSCD can be a useful tool to compare the properties of the evaluated gels if some parameters are varied, and it can be noticed how each parameter is influencing the selected properties [27].

Practically, through this experiment, the gel formulations are optimized considering the evaluated properties highlighted in Figure 3.

Even though there are many cosmetic products with allantoin content, to date, a pharmaceutical product with properties linked to the in-force pharmacopoeial requirements has not been outlined. This study aims to evaluate the influence of the preparation method (mechanical homogenization in a mortar, a method that is usually used in pharmacies, vs. stirring) and the presence/absence of the selected active ingredient (allantoin) considering the properties that are characteristic of a semi-solid formulation, using a mathematical/graphical approach, the SSCD.

## 2. Results and Discussion

### 2.1. The Hydrogel Evaluation Using SSCDs 

**Figure 4:**
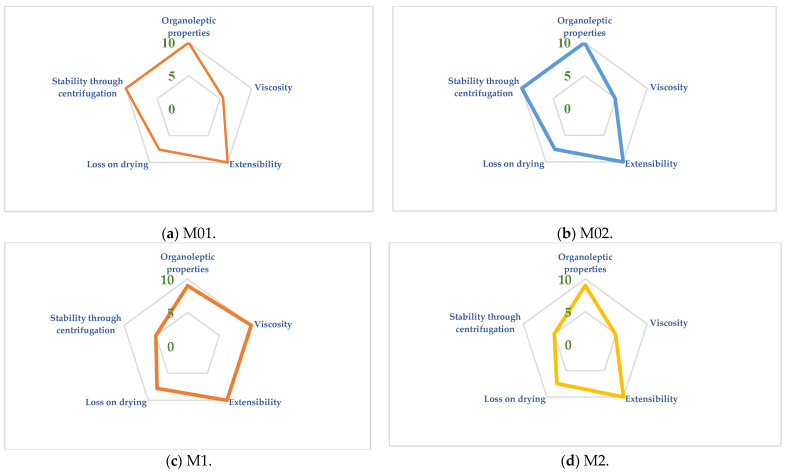
The SSCDs for the blank gels: M01 (**a**), M02 (**b**), M1 (**c**), and M2 (**d**).

The hydrogels can be evaluated through a mathematical and visual method employing SSCDs. The SSCD pictograph visualizes the properties that need improvement or the ones that are already close to the circumference of the pentagon (a fact that implies very good properties). The response of the five different evaluated physicochemical properties can be observed at each corner of the pentagon, depicting a distinct property (Figure 4a–d).

#### 2.1.1. Organoleptic Properties

Considering the organoleptic properties that were summarized with the help of the five parameters experimentally evaluated, it was found that M1 and M2 obtained a score of 9, whilst M01 and M02 obtained the maximum score of 10, a difference that can be explained through the different scores received regarding the homogeneity, as detailed below. Considering an average limit of 5 for the organoleptic properties, all hydrogels exceeded the average selected limit, confirming their very good organoleptic properties. The preparation method did not lead to any modification regarding the organoleptic properties.

##### Homogeneity

In terms of homogeneity, the hydrogel formulations containing allantoin were marked with 1, whilst the blank gels received the maximum value of 2. The blank gels obtained the best homogeneity, a fact that can be explained through the molecular dispersion of the gel-forming ingredient, whilst in the case of the allantoin hydrogels, the active ingredient was suspended, a fact that produced a heterogeneous gel, which is correlated to the dispersion of the active ingredient. 

##### Color

Clear, transparent blank hydrogels were obtained (M01 and M02), and slightly white gels in the case of the allantoin hydrogels (M1 and M2), all of them with a homogenous color and thus receiving the maximum score of 2.

##### Flow through a Tube or Cannula

Considering that all the gels passed smoothly through the cannula with a specified diameter, they all received the maximum score of 2. 

##### Air Absence

**Figure 5:**
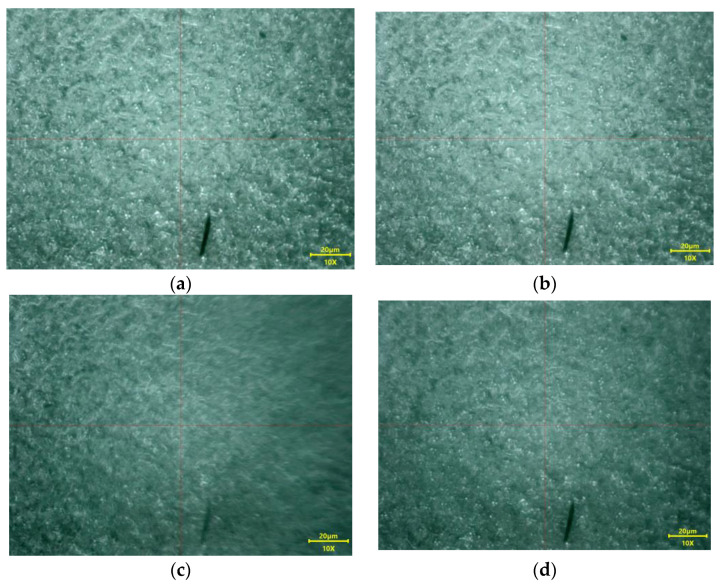
The absence of air bubbles was observed through the microscope method for M01 (**a**), M02 (**b**), M1 (**c**), and M2 (**d**).

Taking into consideration the stability of pharmaceutical products, the lack of air bubbles represents an asset of the formulation. The results show that no air was incorporated during the preparation techniques used, for which a high score of 2 was awarded to each hydrogel developed. The microscope images of the hydrogels developed and the lack of bubbles can be seen in Figure 5a–d.

##### Texture

The samples spread on the glass showed a good texture (as expected), a fact that received the maximum score for all the four gels developed. All the hydrogels received a maximum score of 2.

Considering that SSCDs were used for the development of a lipogel by the team that introduced this mathematical/visual expert system our results will be compared to the ones from this study, in some cases the results from other studies will be evaluated (where available). Nardi-Ricart and collaborators developed an oleogel which was evaluated through the SSCD methodology, which was verified 24 h after its preparation and in stress conditions. It was noticed that the organoleptic properties were evaluated using the same score (7), even after the oleogel was subjected to the stress conditions [27]. In the present study, the radius value registered was higher in both the blank (10) and the allantoin hydrogels (9). 

#### 2.1.2. Viscosity

The gel viscosity ranged between 49 mPa×s for M02 and 116.8 mPa×s for M1. For the viscosity, the lower limit of the v1 was considered 100 mPa×s, considering the targeted properties of the gels. In this regard, the highest radius value was recorded in the case of M1 and the lowest for the M02 hydrogel. For M1 and M01, the target limit value of five was obtained, whilst in the case of M2 and M02, values lower than five were registered. The preparation method influenced the viscosity in the case of the gels prepared in the mortar.

Nardi-Ricart et al. showed that, after the developed oleogel was subjected to stress conditions, the viscosity decreased from 9.2 to 5.4 (in terms of radius). Even if a decrease in this parameter was noticed, the value was maintained higher than the average admitted limit of 5. In our case, two hydrogels highlighted values higher than 5, whilst the other two did not reach this proposed limit [27]. 

#### 2.1.3. Extensibility (Spreadability)

**Figure 6:**
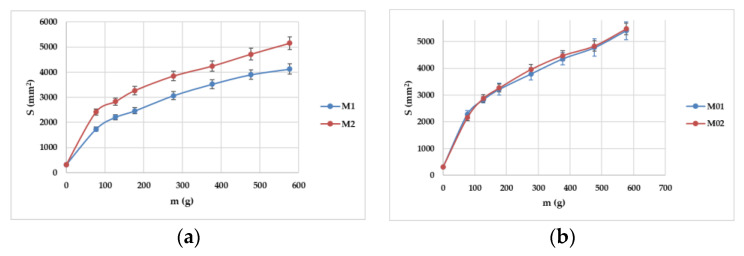
The extensometric curves of the evaluated allantoin hydrogels (**a**) and blank hydrogels (**b**).

Considering that all the gels had a spread surface higher than 1000 mm^2^, they all received the maximum score of 10 (Figure 4a,b). Whilst tracing the extensometric curves, it was noticed that M1 had the lowest final spread surface, whilst the other three hydrogels exhibited close values to 5000 mm^2^ (Figure 6). In this case, the preparation method and the fact that the active ingredient was suspended might be important factors that influence this parameter, as highlighted in the cases of the M1 and M01 hydrogels, for which different values of spreadability were recorded at the end of the experiments.

In the study “A new design for the review and appraisal of semi-solid dosage forms: Semi-solid Control Diagram (SSCD)”, the extensibility was lower than 5 before and after the oleogel was kept in stress conditions [27]. It was noticed that the evaluated semi-solid formulation had higher values after it was kept in stress conditions, a fact that can be explained through the decrease in consistency, which provides better spreadability.

#### 2.1.4. Loss on Drying

All the evaluated gels exhibited radius values higher than the average admitted limit of 5, ranging between 7.44 (M2) and 7.81 (M1). Considering the dispersion media, it was expected that the gels would lose water. Also, the experiment may include a stability test, with the temperature selection whilst drying being an important parameter [27]. 

#### 2.1.5. Stability through Centrifugation

In the blank gels, the homogeneity increases in comparison to the gels where allantoin was included, considering that the allantoin was suspended in the gel base. Their homogeneity was verified both macro- and microscopically. Even though in the case of the allantoin gels the active pharmaceutical ingredient (API) was suspended, they corresponded in terms of homogeneity whilst evaluated visually and at 1000 rpm. Whilst evaluated at 4000 rpm, in the cases of M1 and M2, the allantoin and the hydrogel separated as a result of the increased centrifugation force. The blank gels received the maximum radius score (10) regarding this parameter in comparison to the allantoin hydrogels, which received only 5. 

The stability through centrifugation from the study conducted by Nardi-Ricart et al. showed the same radius value as for the allantoin hydrogels (5), both before and after their oleogel was exposed to the proposed stress conditions; moreover, the blank hydrogels showed better stability, granted by the 10 value, which can be linked to the dispersion type [27].

### 2.2. SSCDs Applied for the Hydrogels’ Evaluation

To evaluate all the parameters, the following indexes were calculated:

- Parameter Index (PI—Table 1). It was noticed that all the gels respected the minimum admitted limit of 0.5 regarding the PI, with the formulation obtaining values higher than 0.8 for all four hydrogels.
- Parameter Profile Index (PPI—Table 1). The PPI ranged between 7.27 for M2 and 8.6 for M01. It can be noticed that the M1, M01, and M02 presented values higher than 8 of these indexes, with the only gels that received a lower score of this index being M2. Even though the hydrogels can be differentiated using this index, all four hydrogels fulfilled the average limit of 5 proposed for this index.
- Good Quality Index (GQI—Table 1). The final and most important index calculated using this method is GQI, whose limit was set at 5. The values of GQI ranged between 5.45 (M3) and 6.45 (M01), all of them respecting the minimum admitted limit of 5. It was noticed that GQI was lower in comparison to PPI, a fact that can be explained by the low value of the reliability factor of 0.75.

Even if all the evaluated parameters receive the maximum score of 10, the maximum value that can be achieved in the case of GQI is 7.5. For a better evaluation, an adjusted GQI (GQIa) was calculated by dividing the values obtained by 7.5 and multiplying by 10 (Table 1). It was noticed that GQIa presents a value very close to the PPI. Another method that might be used to increase the GQI is to increase the number of parameters evaluated to increase the reliability factor (a penetrometry study could be included or the viscosities and tangential stress at different speed levels). In comparison, in the case of SeDeM/SeDeM-SLA diagrams, 12 parameters are evaluated, whilst, in the case of SeDeM-ODT, 15 parameters are evaluated, a fact that leads to a higher reliability factor and higher values of the final calculated quality index (IGCB—Index of Good Compressibility and Bucodispersibility for orodispersible tablets—SeDeM-ODT, f = 0.971; IGC—Index of Good Compressibility in the case of conventional tablets—SeDeM, f = 0.952; or IGF—Index of Good Flow in the case SeDeM-SLA for thesolid–liquid adsorption, f = 0.952. Considering that, for the GQI, the maximum value is 7.5, the minimum admitted value should be lowered to 3.75, due to the low number of parameters evaluated.

The PI, PPI, and GQI recorded in the study conducted by Nardi-Ricart et al. were all lower than the values obtained in this study, but before the oleogel was exposed to stress conditions, all three parameters had values higher than the limits provided by the SSCD expert system. After the oleogel was subjected to stress conditions, the GQI had a value lower than the one admitted of only 4.8, in comparison to this study, where all the evaluated formulations exhibited GQI values higher than 5 [27]. 

### 2.3. Penetrometry (Consistency) Study

**Figure 7:**
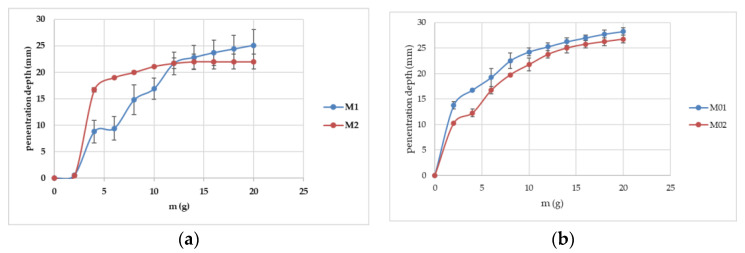
The penetrometric curves of the proposed hydrogels—the allantoin gels (**a**) and the blank hydrogels (**b**).

The penetrometric study represents a useful tool to establish a hydrogel’s consistency. It can be noticed that, when adding the weights, the depth of the penetrometer increases until a plateau is reached. In our case, the plateau was reached after adding weights of 16 g (Figure 7). The depth values expressed in mm ranged between 22 mm for M2 and 28.25 mm for M01 (Figure 7). The allantoin hydrogels tended to be more consistent in comparison to the blank ones, a fact that can be explained through the selected dispersion method (the suspension of the active pharmaceutical ingredient in the hydrogel). This test can be included in the SSCDs as a sixth parameter, considering its utility, a fact that might result in the modification of the reliability factor (f) by increasing it.

In a previously published study conducted by Pintea et al., where seven miconazole hydrogels and seven blank Carbopol hydrogels were developed (the API was suspended in the hydrogel base), values that are close to the ones outlined in this study were obtained. The lowest value registered was 23.2 mm for a blank Carbopol hydrogel and the highest was 29 mm registered for the miconazole hydrogel [28].

### 2.4. Rheology and Flow Behavior

**Figure 8:**
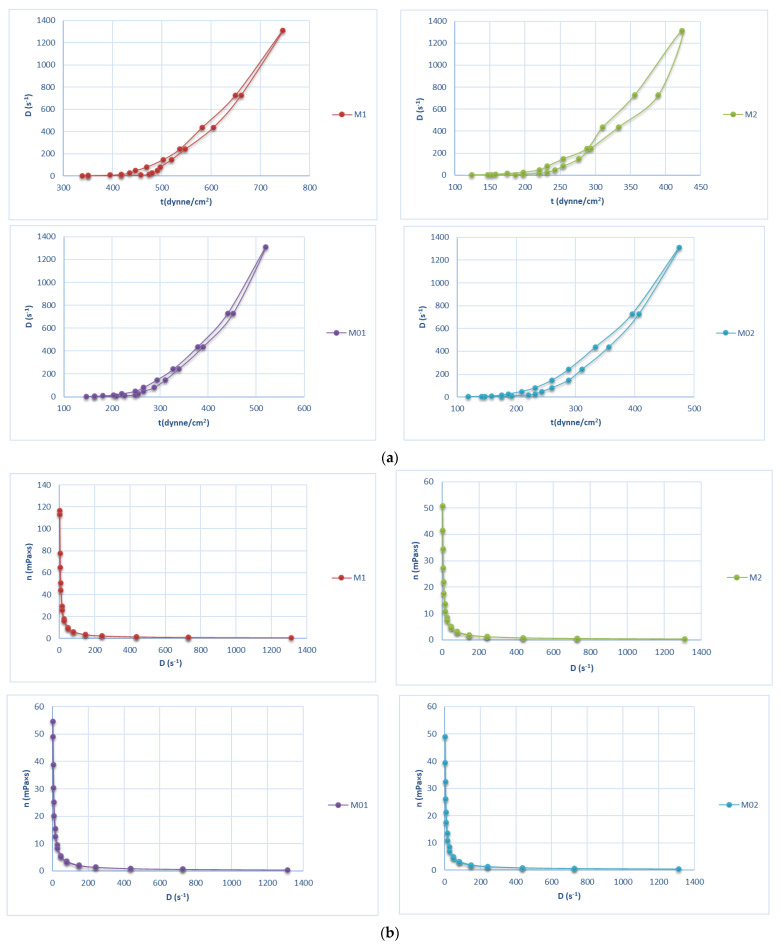
The hydrogels’ flow (**a**) and viscosity (**b**) rheograms for the blank gels and the allantoin hydrogels.

Semi-solids exhibit a complex rheological behavior. For all gels, a pseudoplastic–thixotropic flow can be noticed. By increasing the tangential stress, the flow rate increases, and the viscosity decreases as expected. If the shear rate is increased, the molecule’s reticulation is changed and the product starts to flow. By increasing the speed rate from 1 to 12, the system is destructured, whilst by decreasing the speed rate from 12 to 1, the matrix structure is reorganized. The area between the destructuring curve and the restructuring one is represented by thixotropy. The capability of changing the properties under mechanical properties is an advantage since the systems are more easily extruded and spread. The thixotropy increased in the following order (M2 > M02 > M1 > M01), a fact outlined in Figure 8a. The viscosity curves can be observed in Figure 8b.

The same flow behavior was observed in the studies conducted by Mut et al., where two different gel-forming agents were used (chitosan and hypromellose) to develop Fluconazole hydrogels [29]. The pseudoplastic behavior with thixotropy was also noticed in the study conducted by Karampelas et al., where different cellulose derivatives were used to develop naftifine hydrochloride hydrogels [30].

### 2.5. Drug Content Analysis Using the UV-Vis Spectrophotometric Method

The allantoin content of the hydrogels was in the range of 85–115% for each of the developed hydrogels (M1 and M2), respecting the in-force requirements of the Ph. Eur [31]. The same results were reported by Pintea et al., where 2% Miconazole hydrogels were developed [28], and in the study conducted by Mut et al., where fluconazole hydrogels were developed with a content ranging between 97.61 ± 1.53% and 102.36 ± 2.57% [29].

## 3. Conclusions

Four xanthan gum hydrogels were developed, two that served as blanks and two with the selected ingredient (allantoin), using two distinct methods (one that can be applied in the pharmacy, homogenization using a mortar, and one that can be applied in research laboratories, each of them displaying good pharmacotechnical properties for the gels). Differences occurred between the hydrogels in which allantoin was suspended, but in the future, if the allantoin is dispersed at a molecular level, the organoleptic properties will be improved. The allantoin was suspended in the hydrogel to serve as a gel base that can be used in the future for the incorporation of other APIs to obtain compounding pharmaceutical dermal products. The stability through centrifugation might be also improved by considering the molecular dispersion of the pharmaceutical ingredient in the semi-solid formulation. By considering the GQI and varying the preparation method, the stirring method showed better results in the case of the allantoin hydrogels, whilst in the case of the blank hydrogels (M01 and M02), a small difference was recorded regarding the GQI. The preparation method influenced the evaluated parameters in the case of the allantoin hydrogels, whilst in the case of the blank hydrogels, a smaller difference was recorded. 

## 4. Materials and Methods

### 4.1. Blank and Allantoin Gel Preparation

To prepare the hydrogels, the following ingredients were used: allantoin (Fagron, Trikala, Greece), citric acid (ViVoCHem, Almelo, The Netherlands), xanthan gum (Fagron, Trikala, Greece), glycerol (Chimreactiv, Bucharest, Romania), and a preservative solution (prepared in our laboratory).

The blank hydrogels were prepared at room temperature (20 ± 2 °C) as follows: In the mixture of the glycerol and preservative solution, the citric acid was dissolved. In this dispersion, the xanthan gum was added gradually, ensuring homogenization using trituration or magnetic stirring. In the blank hydrogels, the allantoin was dispersed considering the incorporation using the two selected methods.

Four hydrogels were prepared, two blank (M01 and M02) and two with allantoin (M1 and M2), each of them by the means of two distinctive preparation methods (through stirring, M01 and M1, and trituration in a mortar, M02 and M2). The hydrogels were stirred/mixed in the mortar for 30 min. The composition of the four hydrogels is outlined in Table 2.

### 4.2. The Evaluation of the Proposed Hydrogel Using SSCDs 

The SSCD was traced using the Microsoft Office Excel tool. To obtain the diagrams, the following steps need to be followed: Insert, Chart (Radar). The SSCD is a tool that can be used to characterize a semi-solid formulation. Through this method, the parameter values are converted into a radius (r) [27]. 

The SSCD calculates the following three indexes:

○ Parametric Index (PI).
  PI = nr of parameters > 5/number of total parameters (5) (1)
  where
○ nr of parameters > 5—the number of parameters that are equal to or higher than 5; the limit of acceptance is 0.5, whilst the maximum value that can be obtained is 1.
○ The Parametric Profile Index (PPI) represents the average value of the radius of all parameters.
○ Good Quality Index (GQI) can be calculated using the formula:
  GQI = PPI × f(2)
○ f = reliability factor = polygon area/circle area, which was set at f = 0.75.

The evaluated parameters provide important aspects regarding the hydrogels (in our case). By evaluating these parameters and tracing the SSCDs, a prediction regarding the product quality can be made. The parameters evaluated are organoleptic properties, viscosity, extensibility, water loss, and centrifugation, all of them being detailed in the sections below [27,32].

#### 4.2.1. Organoleptic Properties

This first parameter is composed of another five parameters: homogeneity, color, flow through a tube or cannula, air absence, and texture (each of them receiving a maximum value of 2, so that the cumulated value of the radius is 10, whilst the lowest value that can be registered is 0). Considering that these tests cannot provide quantifiable values, a value of 0, 1, or 2 was granted for each test (detailed in Table 3) (except texture on the glass, to which only 0 and 2 values were ascribed).

#### 4.2.2. Viscosity/Rheology/Flow Behavior

To establish the viscosity and tangential stress, a Rheotest RV viscometer (RHEOTEST Medingen GmbH, Ottendorf-Okrilla, Germany) at 21 ± 2 °C was used, by completing 12-speed levels (increased from 1 to 12 to destructure the gel matrix) and, after reaching the highest level of speed (12), the gel was restructured by decreasing the speed gradually (from 12 to 1). Considering the properties of the gel, a set of coaxial cylinders coded S/S1 was used. A total of 30 mL of sample was measured and introduced in the exterior cylinder (S), whilst the internal cylinder (S1) was attached to the viscometer. The shear rates increased until the point at which they decreased; during this time, the force that was opposed to the rotation was observed (α). The shear rate (t = z×α) and the dynamic viscosity (ƞ = t/D) were calculated, where t is the shear rate, Z represents the cylinder constant, and D denotes the velocity gradient (a constant for each speed and specific to each cylinder) [28].

The value recorded at the highest speed level was considered for this parameter. Considering topical administration, this study aimed to develop low-viscosity gels with a viscosity in the range of 100–1000 mPa×s [27]. To calculate the radius value, the following formula (Equation (3)) was applied.

r = 10 − (v1/100 − v2/100)(3)

where r represents the radius value, v1 shows the reference viscosity = 100 mPa×s, and v2 is the practical value of the viscosity (mPa×s). 

#### 4.2.3. Extensibility (Spreadability)

For the evaluation of the extensibility, the Sune Arbussa/Del Pozo Ojeda extensometer was used. A total of 1 g of gel was placed between two glass plates and weights between 100 and 500 g (from 100 to 100 g) at an interval of 1 min [28]. The etalation surface (π × r^2^) occupied by the gel was noted after each measurement. The radius value was calculated using the following equation (Equation (4)):

r = 10 − (∑1/100 − ∑2/100)(4)

∑1 is the limit suggested for the theoretical surface (100 mm^2^ as the target for ointments and 1000 mm^2^ as the target for hydrogels (fluid semi-solid formulations)), whereas ∑2 denotes the final etalation surface of the evaluated hydrogel (<100 mm^2^, the radius value is 0; if >1000 mm^2^ the radius value is 10) [27].

#### 4.2.4. Loss on Drying

A total of 1 g of each sample was weighed and was heated at 40 °C in an oven (Biobase, Jinan, China) until no modifications were recorded for the gel mass. The amount loss was represented as a percent and it was calculated as follows [33]:

r = 10 − w(5)

where w is the % of mass lost while drying.

#### 4.2.5. Stability through Centrifugation

A total of 5 g of each sample was centrifugated at 1000 rpm and 5000 rpm using a centrifuge (LC-04C ZenithLab centrifuge, ZenithLab, Jiangsu, China) for 15 min. If the samples did not record a separation at 1000 rpm but occurred at 5000 rpm, a value of 5 for the radius was granted, whilst if the samples did not separate at 5000 rpm, a value of 10 was awarded. If the sample separated at 1000 rpm, a 0 value for the radius was granted [27,34].

### 4.3. Penetrometry (Consistency) Study

To conduct the penetrometric study, a penetrometer was used, which had a conic metal part attached to a bar and by free falling settled on the center of the surface of the gel. An amount of gel was placed in a plastic box (height 30 mm and diameter 50 mm) and the penetration from the surface of the gel to the peak of the cone was measured. Weights that were in the range of 2–20 g (from 2 to 2 g) were added to the cone, noticing the distance (in mm) after every measurement [28,34].

### 4.4. Drug Content Analysis Using the UV-Vis Spectrophotometric Method

To establish the concentration in the gel, a Shimadzu UV-Vis spectrophotometer (Mettler Toledo, Columbus, OH, USA) was used. The amount of the API was assayed with the help of the UV-spectrophotometric method at 208 nm. The linearity and specificity of the method were evaluated [31]. A stock solution of 1 mg/mL was prepared by dissolving 0.1 g of allantoin in 100 mL of phosphate buffer, at a pH = 6.8. From the stock solution, five dilutions ranging from 1 to 25 µg/mL were made. To verify the specificity, a blank gel was evaluated at the same pre-selected wavelength, where no interference was detected. 

## Figures and Tables

**Table 1:** The PI, PPI, and GQI/GQIa of the developed hydrogels.

Index	M1	M2	M01	M02
PI	1	0.8	1	0.8
PPI	8.36	7.27	8.6	8.5
GQI	6.27	5.45	6.45	6.38
GQIa	8.36	7.26	8.6	8.51

**Table 2:** The compositions of the blank and allantoin hydrogels.

Ingredient	Role	Formulation Code			
		M01	M02	M1	M2
Allantoin	Active ingredient	-	-	1	1
Xanthan gum	Gel-forming agent	1	1	1	1
Citric acid	Keratin softener	1	1	1	1
Glycerol	Humectant	5	5	5	5
Preservative solution	Preservative/Dispersion media	ad 100	ad 100	ad 100	ad 100

**Table 3:** The limit values for the five parameters considered when evaluating the organoleptic properties for 1 g of sample.

Parameters/Test	Limit Values		
	0	1	2
Homogeneity (on a glass plate)	Discontinuities that can be noticed visually (inhomogeneous dispersion)	Small discontinuities can be noticed with a microscope (partially homogenous)	No physical discontinuities can be noticed (homogenous)
Color (visual evaluation)	Different shades can be noticed	Non-uniform parts are almost imperceptible	Uniform
Flow through a cannula (Ø = 4.8 mm; length of 40 mm, using manual force)	Excessive force is required for flowing	Flows with difficulty	Passes smoothly
Air absence (visual evaluation)	Air bubbles are observed by visual evaluation	Air bubbles can be noticed with the help of a microscope	Air absence through microscope visualization
Texture (on glass)	Difficult to spread	-	It can spread properly

## Data Availability

All data and materials are available on request from the corresponding author. The data are not publicly available due to ongoing research using a part of the data.
